# The Pattern of AQP4 Expression in the Ageing Human Brain and in Cerebral Amyloid Angiopathy

**DOI:** 10.3390/ijms21041225

**Published:** 2020-02-12

**Authors:** Raisah Owasil, Ronan O’Neill, Abby Keable, Jacqui Nimmo, Matthew MacGregor Sharp, Louise Kelly, Satoshi Saito, Julie E. Simpson, Roy O. Weller, Colin Smith, Johannes Attems, Stephen B. Wharton, Ho Ming Yuen, Roxana O. Carare

**Affiliations:** 1 Faculty of Medicine, University of Southampton, Southampton SO16 6YD, UK; raisah.owasil@icloud.com (R.O.); ron1e11@southamptonalumni.ac.uk (R.O.); A.C.Keable@soton.ac.uk (A.K.); jtn1g13@soton.ac.uk (J.N.); m.t.sharp@soton.ac.uk (M.M.S.); L.Kelly@soton.ac.uk (L.K.); saitou.satoshi.43m@kyoto-u.jp (S.S.); row@soton.ac.uk (R.O.W.); H.M.Yuen@soton.ac.uk (H.M.Y.); 2 Sheffield Institute for Translational Neurosciences, University of Sheffield, Sheffield S10 2HQ, UK; julie.simpson@sheffield.ac.uk (J.E.S.); s.wharton@sheffield.ac.uk (S.B.W.); 3 Academic Neuropathology, Centre for Clinical Brain Sciences, University of Edinburgh, Edinburgh EH16 4SB, UK; col.smith@ed.ac.uk; 4 Translational and Clinical Research, Newcastle University, Newcastle NE2 4HH, UK; johannes.attems@ncl.ac.uk

**Keywords:** aquaporin 4, cerebral amyloid angiopathy, white matter hyperintensities, grey matter, white matter

## Abstract

In the absence of lymphatics, fluid and solutes such as amyloid-β (Aβ) are eliminated from the brain along basement membranes in the walls of cerebral capillaries and arteries—the Intramural Peri-Arterial Drainage (IPAD) pathway. IPAD fails with age and insoluble Aβ is deposited as plaques in the brain and in IPAD pathways as cerebral amyloid angiopathy (CAA); fluid accumulates in the white matter as reflected by hyperintensities (WMH) on MRI. Within the brain, fluid uptake by astrocytes is regulated by aquaporin 4 (AQP4). We test the hypothesis that expression of astrocytic AQP4 increases in grey matter and decreases in white matter with onset of CAA. AQP4 expression was quantitated by immunocytochemistry and confocal microscopy in post-mortem occipital grey and white matter from young and old non-demented human brains, in CAA and in WMH. Results: AQP4 expression tended to increase with normal ageing but AQP4 expression in severe CAA was significantly reduced when compared to moderate CAA (*p* = 0.018). AQP4 expression tended to decline in the white matter with CAA and WMH, both of which are associated with impaired IPAD. Adjusting the level of AQP4 activity may be a valid therapeutic target for restoring homoeostasis in the brain as IPAD fails with age and CAA.

## 1. Introduction

Accumulations of amyloid-β (Aβ) and tau proteins in the brain are well-recognised pathological features of Alzheimer’s disease [1]. Fluid also accumulates in the brain with age and Alzheimer’s disease as seen on MRI as dilated perivascular spaces in the white matter and by white matter hyperintensities (WMH). Why fluid accumulates in these sites is still under debate, but advancing age and CAA correlate with dilated perivascular spaces and with WMH [2]. Accumulation of Aβ and of fluid in the brain suggests that there may be loss of homoeostasis in Alzheimer’s disease due to failure of elimination of these components and other solutes from the brain.

In the absence of lymphatic vessels in the brain parenchyma, fluid and solutes drain from the brain to cervical lymph nodes along basement membranes in the walls of cerebral capillaries and arteries [3]. The route of drainage is along basement membranes in the walls of capillaries and, most noticeably, along basement membranes of smooth muscle cells in the tunica media of cerebral arteries [4,5]; it has therefore been named the Intramural Peri-Arterial Drainage (IPAD) pathway [6]. As arteries age, the efficiency of IPAD for the elimination of soluble Aβ from the brain is impaired and even more reduced by the deposition of insoluble Aβ in basement membranes of the IPAD pathways in cerebral amyloid angiopathy (CAA) [7,8].

In this paper, we approach the accumulation of fluid in the white matter in Alzheimer’s disease and CAA from a different angle. We examine how levels of expression of Aquaporin 4 (AQP4) associated with astrocytes in the brain change in relation to the patient’s age and the presence of CAA, as well as WMH. AQP4 appears to be involved in the removal of oedema fluid from the brain in a variety of disorders and enhancing AQP4 expression could be a therapeutic strategy for reducing dilated perivascular spaces and WMH associated with advancing age and Alzheimer’s disease.

Aquaporin 4 (AQP4) is the predominant water channel in mammalian brain expressed in astrocyte endfeet bordering the ventricles, subarachnoid space, blood vessels, glial processes of the granule cell layer in the cerebellum [9] and to a lesser extent in adluminal and abluminal endothelial cell membranes [10,11]. In humans and mice, the distribution of AQP4 is predominantly localised to astrocytic endfeet adjacent to cerebral vasculature. However, in humans a higher proportion of AQP4 is also expressed in astrocytic cell membranes facing the parenchyma [12]. The expression of AQP4 is influenced by anatomical location [9,13,14,15] and by disease. For example, in experimental models of ischaemia or in the deep white matter of post stroke dementia, there is a redistribution of AQP4 from astrocyte end feet to the astrocytic cell bodies [16,17].

A key function of AQP4 appears to be the regulation of water transport across astrocytic cell membranes. In diseases that alter brain water homeostasis such as brain tumours [18,19], stroke [20,21] and traumatic brain injury [22], AQP4 expression is increased. This is thought to be associated with oedema resolution [23]. Mice deficient in AQP4 appear to be less affected by cytotoxic oedema [24] but more susceptible to vasogenic and hydrocephalic oedema [25,26].

White matter hyperintensities (WMH) [2] and abnormalities in fluid homeostasis in the white matter are frequent radiological observations in neurodegenerative diseases. White matter is highly susceptible to oedema after acute injury to the central nervous system (CNS) [27,28,29] and WMH are a common finding on computer tomography (CT) or magnetic resonance imaging (MRI) in the elderly, in stroke, dementia and cerebral amyloid angiopathy (CAA) [30]. Dilated perivascular spaces visible on MRI have been described in the white matter of patients with Alzheimer’s disease (AD) or CAA [31]. Previous studies have shown that AQP4 expression is altered in mouse models of AD and humans with AD [32,33,34] and have indicated a link between AQP4 expression and amyloid burden [35]. A recent study showed elevated expression of AQP4 in the grey matter from autopsied temporal lobes from eight patients with Alzheimer’s disease (AD) [36]. However, it is not clear if alterations in AQP4 expression in the grey matter are accompanied by a similar pattern in the expression of AQP4 in the white matter or whether AQP4 expression is linked to severity of CAA.

Due to the dynamic role that astrocytes play in neurodegenerative diseases [37,38], in this study we analysed how the expression of AQP4 in the white and grey matter changes in the occipital cortex obtained from young and old non-demented humans. We then investigated whether these changes were altered in neurodegenerative diseases by comparing AQP4 expression in (a) the white matter and grey matter of occipital cortex obtained from human cases of CAA with (b) white matter obtained from cases with WMH and (c) white and grey matter from non-demented age matched controls. Finally, we investigated how the severity of neurodegenerative disease affects AQP4 expression by comparing white and grey matter of occipital cortex obtained from human cases of moderate or severe CAA.

Dilated perivascular spaces have not been observed in the cerebral cortex, even when there is pathology present [39] but they are common in the white matter of patients with CAA [31,40]. Considering the role of AQP4 as a water channel and that WMH and dilated perivascular spaces reflect an excess of fluid in the parenchyma, here we test the hypothesis that expression of AQP4 increases in the grey matter and decreases in the white matter with onset of CAA when compared to age matched controls. As the physiological capacity of AQP4 for clearing fluid is exceeded, we suggest that the expression of AQP4 is decreased in white matter with WMH, compared to the white matter of young and old non-demented cases.

## 2. Results

### 2.1. The Pattern of Immunostaining for AQP4 Is Different in the Grey Compared to White Matter and Changes with Age and with Severity of CAA

**Figure 1:**
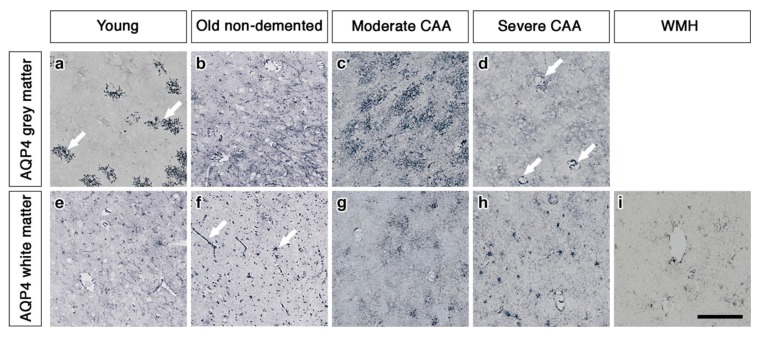
Pattern of AQP4 immunostaining in the parenchyma of young, non-demented aged control, CAA and WMH brains. In young brains, individual AQP4 positive arborized astrocytes (arrows) are scattered in the cortex (**a**) with faintly stained small cell bodies in the white matter (**e**). In old non-demented brains, immunostaining of AQP4 appears granular and outlining processes, no longer distinguishing individual astrocytes in either grey (**b**) or white matter. Immunoreactivity in the white matter appears to outline blood vessels (arrows) (**f**). In moderate CAA, immunostaining reveals highly arborized AQP4 positive astrocytes, with staining of both cell bodies and processes in the grey matter (**c**). Only punctate sparse staining is observed in the white matter (**g**). In severe CAA, immunostaining for AQP4 is sparse and weak in both grey matter (**d**) and white matter (**h**) but mainly in a perivascular location (arrows) in the grey matter (**d**). In WMH brains, immunostaining of AQP4 is very weak, with few faintly stained processes (**i**). Scale bar 100 µm.

**Figure 2:**
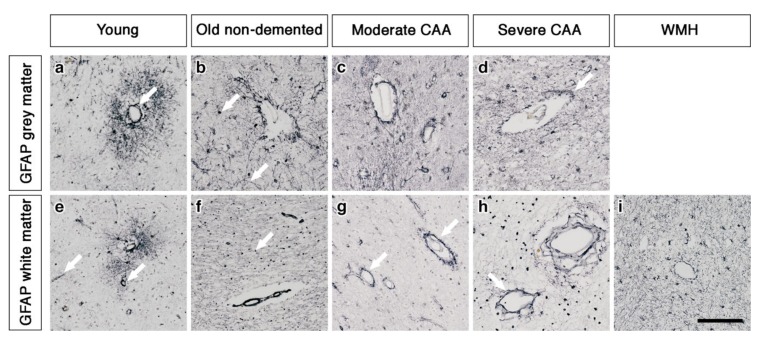
The pattern of immunostaining for GFAP associated with the blood vessels of grey and white matter. In young brains, GFAP immunostaining reveals highly arborized astrocytes and profiles of blood vessels of all sizes in both grey and white matter (arrows) (**a** and **e**). In old non-demented brains, the GFAP immunostaining appears intense in the grey matter, highlighting fine processes and cell bodies (arrow) (**b**) but granular (arrow) and mostly in perivascular locations in the white matter (**f**). In the grey matter of moderate CAA brains, the immunostaining of GFAP shows intensely stained processes surrounding the vessels (**c**) but in the white matter is confined to the perivascular locations (arrows) (**g**). In severe CAA brains, GFAP immunostaining is intense and localised to the blood vessels of both grey (arrow) (**d**) and white matter (arrow) (**h**) with small cell bodies also present in the white matter (**h**). In WMH brains the GFAP immunostaining is relatively intense, highlighting fine processes and cell bodies in the white matter and surrounding vessels (**i**). Scale bar 100 µm.

In the grey matter from young brains, immunostaining revealed individual AQP4 positive arborized astrocytes scattered in the cortex (Figure 1a), particularly concentrated at the grey-white matter border. Fewer AQP4 positive astrocytes were observed in the white matter (Figure 1e). Glial fibrillary acidic protein (GFAP) immunostaining revealed highly arborized astrocytes surrounding the profiles of blood vessels of all sizes (Figure 2a,e). In brains from old non-demented controls, immunostaining of AQP4 appeared fibrillary or granular and no longer distinguished individual astrocytes. The AQP4 immunoreactivity appeared to reveal the profiles of blood vessels (Figure 1b,f). GFAP immunostaining was very intense, particularly in fine processes and cell bodies (Figure 2b,f). In grey matter from brains with moderate CAA, immunostaining revealed highly arborized AQP4 positive astrocytes, with staining of both cell bodies and processes. There was no apparent relationship with the profiles of blood vessels and a reduction of AQP4 immunoreactivity at the grey–white matter border was observed. Only punctate sparse staining was observed in the white matter (Figure 1c,g). GFAP immunostaining showed the profile of many astrocyte processes and a perivascular location in the grey matter. In the white matter, the high density of stained processes observed with AQP4 immunostaining was not present with GFAP immunostaining and the staining with GFAP was mainly perivascular (Figure 2c,g).

In brains with severe CAA, the immunostaining for AQP4 was sparse and weak (Figure 1d,h) and mainly in a perivascular location in the grey matter. In contrast, GFAP immunostaining was intense and localised to perivascular locations of both grey and white matter (Figure 2d,h). In white matter hyperintensities the immunostaining for AQP4 was very weak in comparison to the strong GFAP staining (Figure 1i and Figure 2i).

### 2.2. Double Immunostaining

**Figure 3:**
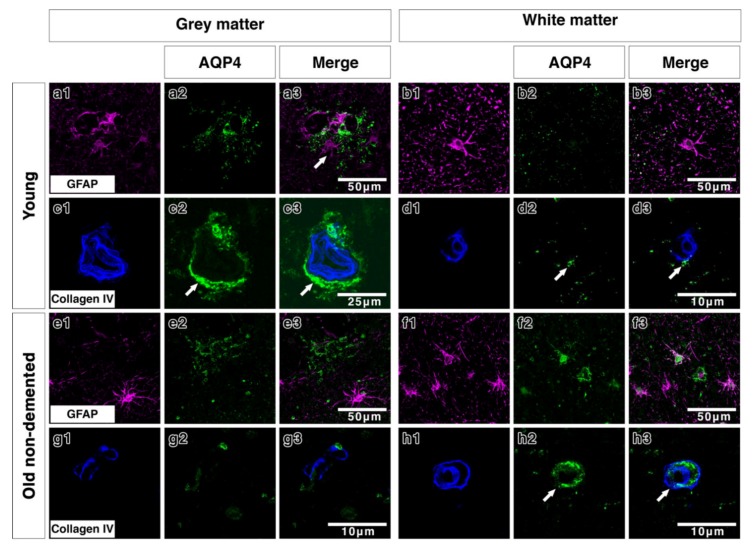
Double immunofluorescence for AQP4 (green) and GFAP (magenta) or collagen IV (blue) in young and old-demented brains. In grey matter from young brains, AQP4 appears highly arborized in a perivascular location (arrows **c2,c3**) with some degree of association with GFAP (**a1–a3** and **c1–c3**). In white matter from young brains, AQP4 immunostaining is faint, with very small degree of co-expression with the wide distribution of GFAP-immunostained cells and fine processes (**b1–b3**) and with only little relation to collagen IV (arrows) (**d1–d3**). In the grey matter of old non-demented brains, AQP4 immunostaining shows a diffuse fibrillary pattern with small cell bodies in comparison to the larger cell bodies and processes immunostained with GFAP, with almost no association with GFAP positive astrocytes (**e1–e3**) and with very sparse perivascular distribution (**g1–g3**). In the white matter of old non-demented brains, AQP4 is associated with GFAP positive cell bodies (**f1–f3**) and in a perivascular location as shown by co-expression with collagen IV in the wall of the blood vessel (arrows) (**h1–h3**).

**Figure 4:**
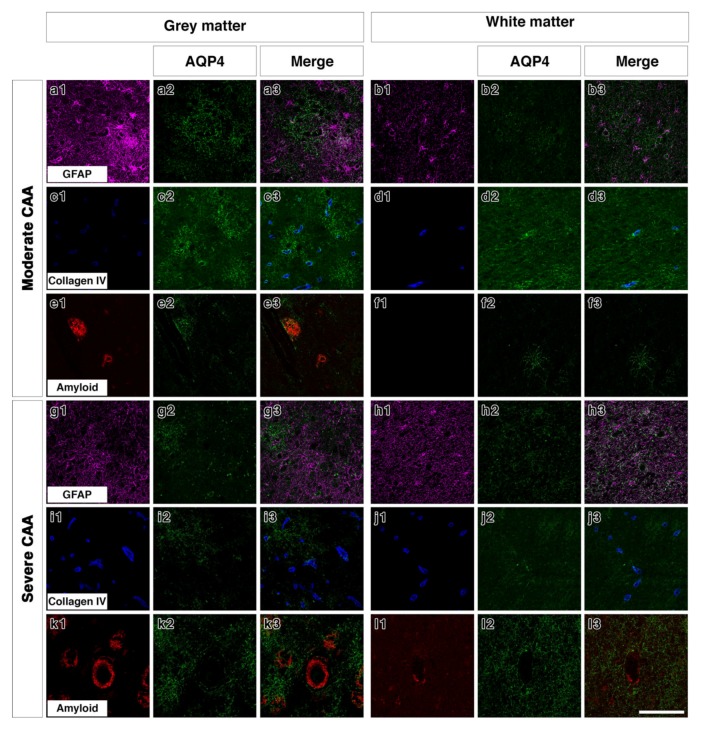
Double immunofluorescence for AQP4 (green) with GFAP (magenta), collagen IV (blue) or Aβ (red) in moderate or severe CAA. In the grey matter of moderate CAA, both AQP4 and GFAP immunostaining appears intense with AQP4 more diffuse (**a1–a3**). AQP4 appears in a perivascular location as shown by the association with collagen IV (**c1–c3**) but also appears associated with the presence of Aβ (**e1–e3**). In the white matter of moderate CAA, AQP4 immunostaining appears fainter (**b1–b3**), with some close relation to collagen IV (**d1–d3**). No Aβ immunostaining was detected in the white matter (**f1–f3**). In the grey matter of severe CAA, immunostaining of GFAP appears more intense than that of AQP4 (**g1–g3**). Immunostaining of AQP4 (**g2**) appears reduced when compared to that seen in the grey matter of moderate CAA (**a2**) but can be observed in a perivascular location, as seen by the relation to collagen IV immunostaining (**i1–i3**). Immunostaining of AQP4 can also be seen in close proximity to blood vessels laden with Aβ (**k1–k3**). In the white matter of severe CAA, the immunostaining of AQP4 appears diffuse with some punctate cell bodies stained and little perivascular distribution (**h1–h3** and **j1–j3**). Granular AQP4 immunostaining can be seen surrounding blood vessels containing some Aβ (**l1–l3**). Scale bar 100 µm.

Double immunostaining in young brains revealed the association of AQP4 with GFAP positive astrocyte cell processes in discrete areas (Figure 3(a1–a3)), particularly near collagen IV positive blood vessels in the grey matter (Figure 3(c1–c3)). In the white matter of young brains the AQP4 was punctate and faint with a small degree of coexpression with GFAP and perivascular collagen IV (Figure 3(b1–b3,d1–d3)). In old control brains, double immunostaining revealed a more diffuse distribution of AQP4 (Figure 3(e2)) in the grey matter. Very little AQP4 was closely associated with GFAP positive cell bodies (Figure 3(e3)) and blood vessels in the grey matter (Figure 3(g1–g3)). In the white matter of old non demented brains, there was a close association of AQP4 with GFAP and with collagen IV associated with blood vessels (Figure 3(f1–f3,h1–h3)). In moderate CAA, there was little or no co-expression observed between AQP4 and GFAP in both grey (Figure 4(a1–a3)) and white matter (Figure 4(b1–b3)) and immunostaining for collagen IV demonstrated some perivascular localization of AQP4 (Figure 4(c1–c3,d1–d3)). AQP4 did co-localise with the amyloid deposits that were mainly observed in the grey matter (Figure 4(e1–e3)). No amyloid deposits were observed in the white matter (Figure 4(f1–f3)). In severe CAA, the immunostaining for GFAP was far more intense and occupied all of the field of view (Figure 4(g1–h1)), in comparison to the small perivascular immunostaining for AQP4. Collagen IV immunostaining confirmed the presence of the weak punctate AQP4 immunostaining around but not exclusively limited to blood vessels especially in the grey matter (Figure 4(i3,j3)). The AQP4 staining was arborized and diffuse in the areas of vascular amyloid in both grey and white matter (Figure 4(k3,l3)).

### 2.3. AQP4 Expression Is Altered with Severity of CAA

**Figure 5:**
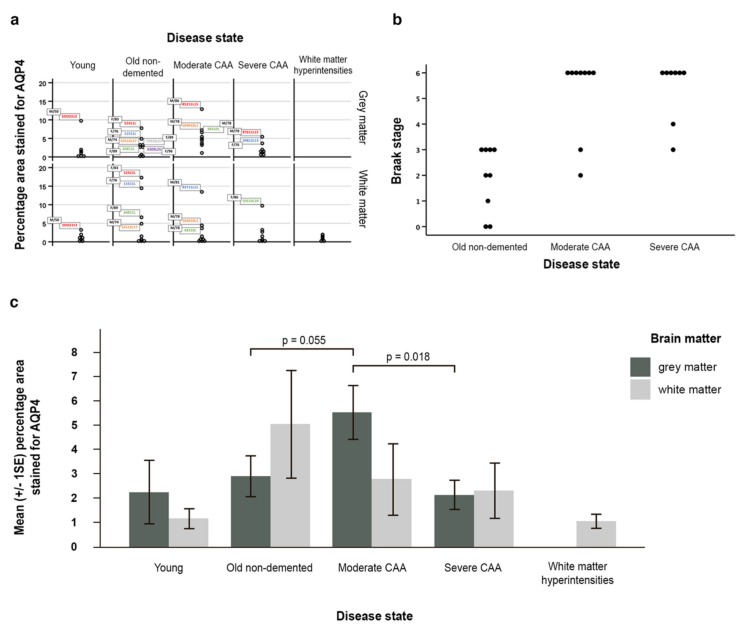
Graphical representation of the data and statistical analyses. (**a**) all data demonstrating skeweness; (**b**) a scatter pot (with identical values stacked) reflecting the relationship between Braak stage and disease state; (**c**) graph with the mean (±SE) percentage area of AQP4 immunostaining for grey and white matter in young, old non-demented, moderate and severe CAA, as well as white matter hyperintensities. In moderate CAA, the percentage area stained for AQP4 was higher in the grey matter compared to the white matter and higher than the grey matter in old non-demented age matched controls (*p* = 0.055). When compared to moderate CAA, the grey matter in severe CAA showed a decrease in the percentage area stained for AQP4 (*p* = 0.018). Significance level is set at 0.0125 by the Bonferroni method for grey matter due to multiple comparisons.

The outcome variables percentage area stained for AQP4 in grey and in white matter were both positively skewed (Figure 5a). No particular pattern emerged from the outliers regarding age and gender. The outliers in the young and in the old non-demented group seemed to have higher percentage areas stained for AQP4 in both grey in white matter; however, this is not the case in the two CAA groups.

Braak stage and disease state were ordinal variables and skewed. Spearman’s rank correlation coefficient showed that the disease state and Braak stage were significantly related (*n* = 26, rho = 0.704, *p* < 0.001) indicating a positive strong correlation between disease state and Braak stage (Figure 5b).

AQP4 expression was increased in grey and white matter with normal ageing. In the white matter, AQP4 expression decreased with disease, with the highest expression in old aged controls, followed by CAA and WMH. In the grey matter, AQP4 expression increased from young to old non-demented, then peaked at moderate CAA and then decreased in severe CAA (Figure 5c, Table S1a).

Although not statistically significant (*p* = 0.319) the average percentage area stained for AQP4 was higher in the grey matter (2.21%) than in the white matter (1.13%) in young brains. In contrast, in the old non-demented brains, this was higher in the white matter (5.01%) when compared to the grey matter (2.86%), but this difference was also not statistically significant (*p* = 0.192) (Table S1b). When compared to young brains, the white matter of old non-demented brains showed a 3.88% increase in mean percentage area stained for AQP4 (Figure 5c, Table S1(c2)), and 0.48% in terms of the median difference (Table S1(c1)).

In moderate CAA, the average percentage area stained for AQP4 was higher however not significant in the grey matter (5.49%) compared to the white matter (2.76%) (*p* = 0.175, Table S1b) and higher than the grey matter in old non-demented age matched controls (5.49% vs 2.86%) (*p* = 0.055) (Figure 5c, Table S1(c2)). Although not significant (*p* = 0.895), in severe CAA brains the percentage area stained for AQP4 was higher in the white matter (2.27%) when compared to the grey matter (2.09%) (Figure 5c, Table S1b). When compared to moderate CAA, the grey matter in severe CAA showed a significant decrease in the percentage area stained for AQP4 (2.09% vs 5.49%) (*p* = 0.018 at 5% significance level) (Figure 5c, Table S1(c2)), but not significant marginally after adjusting for multiple comparisons. The white matter also showed a decrease in the percentage area stained for AQP4 but this was not significant (2.27% vs 2.76%, *p* = 0.807) (Table S1(c2)).

The univariate linear regression model on percentage area stained for AQP4 in grey matter showed that disease state and Braak stage were both significantly related to the outcome (*p* = 0.030 and 0.005 respectively, Table S1d). Disease state remained as a significant factor (*p* = 0.013) after adjusting for Braak stage, age and sex. The key adjusted differences amongst the disease states were when moderate CAA compared to old non-demented controls (*p* = 0.010, Table S1d) and when compared to severe CAA (*p* = 0.013, Table S1e). Braak stage was no longer a statistically significant factor after adjusting for the 3 covariates. Regression model without adjusting for age and sex was also developed and the results were similar to the full model (Table S1d,e). We did not observe any significant results in the regression models on white matter.

## 3. Discussion

This study shows the quantitative differences in the percentage area immunostained for AQP4 in grey and white matter from young, old non-demented, CAA and WMH brains. We demonstrate that AQP4 immunostaining in the grey matter of young brains outlines clearly astrocyte cell bodies and processes. In brains from old, non-demented individuals, the pattern of immunostaining is granular and in the white matter outlines the profiles of blood vessels. A power calculation was not performed at the beginning of this study, as the number of eligible brain samples we could access was very limited. Moreover, we have taken a conservative approach by adjusting the statistical significance level with the Bonferroni method when comparing the disease states, hence some of our key findings are not statistically significant.

In the grey matter of moderate CAA, AQP4 outlines individual cell bodies and processes of astrocytes. When compared to the grey matter of severe CAA brains, the expression of AQP4 was higher in the moderate CAA cases compared to age-matched controls, supporting previous reports of astrogliosis in CAA, where MMP2 positive astrocytes were observed surrounding the CAA-related haemorrhages [41].

We observed a trend towards increased expression of AQP4 with ageing and in the grey matter of moderate CAA compared to non-demented age matched controls. In contrast, when analysing the white matter, the highest expression was observed in the non-demented age matched controls, followed by CAA and WMH. Our results are in agreement with recent studies that showed an increased expression of AQP4 with advancing age and in CAA, although these studies did not perform separate analyses for the grey and white matter [42] and did not include white matter hyperintensities [43]. Individual processes of astrocytes stained by AQP4 were observed only in moderate CAA. Loss of perivascular AQP4 is associated with increased severity of Aβ plaques [42], and recent work proposes that the distribution of AQP4 around plaques is associated with formation of a glial net surrounding the plaque, recruitment of microglia, with a role in the partial protection of nearby neurons from the deleterious effects of Aβ aggregates [44].

When AQP4 was compared to immunostaining for GFAP, the striking differences were observed in old non-demented age matched controls and in CAA. In the old non-demented age matched controls, the main differences between the pattern of immunostaining for AQP4 and GFAP in the grey matter were that AQP4 immunostaining showed processes and a granular appearance not associated with blood vessels, whereas GFAP was intensely outlining blood vessels and in very fine processes. In moderate CAA, the immunostaining for AQP4 was intensely arborized, with no obvious relation to the blood vessels in either grey or white matter, whereas GFAP was confined mainly to blood vessels. There was obvious loss of AQP4 immunostaining in both grey and white matter of severe CAA cases compared to moderate CAA or old non demented cases. In contrast, the immunostaining for GFAP in severe CAA was similar to that of moderate CAA, with a distribution mainly in a perivascular location. These results suggest that GFAP indeed reflects a generalised increase in the function of astrocytes with ageing and with CAA, whereas the water channel AQP4 increases in moderate CAA but is almost absent in severe CAA. The discrepancy between the patterns of immunostaining for AQP4 and GFAP was very obvious in cases of WMH. While AQP4 was very faint with only a few processes immunostained, the GFAP-positive astrocytes had many processes and small cell bodies, with perivascular immunostaining. This morphological discrepancy may reflect the failed function of AQP4 to maintain water balance in WMH while astrocytes are upregulated in their function overall. The phenotype of astrocytes changes with CAA, as reflected by changes in GFAP markers [32].

The double immunofluorescence staining for AQP4 and GFAP or Collagen IV demonstrated that in the young grey matter AQP4 and GFAP were distributed in a very similar pattern, whereas in the old non-demented controls there appeared to be more GFAP staining in both grey and white matter. In young and old non-demented controls, AQP4 was distributed in a perivascular pattern as demonstrated by collagen IV immunostaining, confirming previous reports [32]. This perivascular distribution was maintained in moderate CAA, in which AQP4 immunostaining was highly arborized in the grey matter, but was lost in the grey matter of severe CAA. While we did not perform any biochemical analyses, our findings are in agreement with previous reports that demonstrated an overall reduction in the gene expression of AQP4 in severe CAA [32].

Previous studies focused on the elimination of fluid and solutes from the brain have demonstrated that the major drainage pathway is along basement membranes in the walls of cerebral capillaries and basement membranes surrounding smooth muscle cells in the tunica media of cerebral arteries. This is the Intramural Peri-Arterial Drainage (IPAD) pathway that is effectively the drainage route for fluid and solutes from the brain to cervical lymph nodes [45,46]. Our present results reflect the dynamic changes that occur within the brain parenchyma with age and CAA by recording the expression and localization of AQP4 channels with increasing age and CAA. The increased expression of AQP4 in the grey matter accompanying moderate CAA probably reflects the increased need for reactive astrocytes to clear excess fluid resulting from interference with fluid drainage that accompanies CAA. Responses within the brain through expression of AQP4 appear to be lost with advancement of disease to severe CAA. A clear picture of how the pattern of immunocytochemistry of AQP4 changes in the grey and white matter in health and disease will advance our knowledge of how best to target AQP4 for rebalancing levels of fluid and solutes in the brain in CAA and Alzheimer’s disease [47].

## 4. Materials and Methods

### 4.1. Experimental Subjects

Brain tissue cohort: tissue from younger individuals (≤50 years old) was supplied by the MRC funded Edinburgh Brain Bank (Ethics REC 16/ES/0044), old (70–96 years old) and CAA (62–99 years old) tissue was supplied by Newcastle Brain Tissue Resource (Ethics REC 08/H0906/136) and tissue correlating to WMH (78–91 years old) was supplied by the Cognitive Function Ageing Study (CFAS), Sheffield (Ethical approval 15/SW/0246) (Table 1). Cases from Newcastle were diagnosed according to internationally used criteria including neuritic Braak stages [48], Thal amyloid phases [49], CERAD scores [50], NIA-AA guidelines [51] and McKeith criteria for Lewy body disease [52]; these cases showed varying degrees of AD pathology with no Lewy Body pathology.

The severity of CAA in the occipital lobe was graded according to the system proposed by Vonsattel and colleagues [53] which refers to severity of CAA in individual blood vessels: “mild”, amyloid is restricted to the tunica media without significant destruction of smooth muscle cells; “moderate”, the tunica media is replaced by amyloid and is thicker than normal; “severe”, extensive amyloid deposition with focal wall fragmentation or even ‘double barrelling’ of the blood vessel wall, microaneurysm formation, fibrinoid necrosis and leakage of blood through the blood vessel wall [54]. To provide a better estimation of the combined severity of CAA across the entire histological section from the occipital lobe, we scored CAA according to the method described by Love and colleagues [55]: score 1 refers to scant amyloid-β (Aβ) deposition in the vessel wall, while score 2 refers to some circumferential and score 3 to widespread circumferential Aβ deposition, respectively. Parenchymal and meningeal CAA are separately scored and scores are added to provide an overall score. The presence of Aβ deposition in capillary walls (i.e., capillary CAA) is scored as 1 and added to the sum of parenchymal and meningeal scores. Based on the presence of capillary CAA, CAA can be classified into type 1 where capillary CAA is present and type 2 which lacks capillary CAA [56]. For this study, cases with CAA scores [55] below 6 were staged as low/moderate CAA, and cases with scores of 6 or higher were considered to have severe CAA (see Table 1). Moderate and severe CAA cases were diagnosed using a staging system which assesses meningeal, parenchymal and capillary CAA separately [57]. None of these cases was diagnosed with CAA during life.

Cases from the MRC Edinburgh Brain Bank had no neurological disease during life and no significant neuropathological changes post mortem. We have excluded any cases with arteriolosclerosis/lipohyalinosis from this cohort. Tissue sampling from formalin-fixed coronal cerebral slices was guided by post-mortem MRI scans to identify WMH, as previously described [58], and the lesion/non-lesional status of the sampled blocks confirmed by Luxol fast blue stain for myelin loss and immunohistochemisty to the microglial marker CD68. All samples were collected and prepared in accordance with the National Research Ethics Service approved protocols. 

### 4.2. Assessment of AQP4 Expression by Immunohistochemistry

All groups were qualitatively and quantitatively assessed for AQP4 and qualitatively assessed for GFAP. AQP4 was both qualitatively and quantitatively assessed in all disease states using anti-aquaporin 4 (rabbit polyclonal, 1:200;, Santa Cruz Biotechnology, Dallas, TX, USA). We also qualitatively assessed astrocytes using anti-GFAP in all disease states (rabbit polyclonal, 1:500; Agilent Dako, Santa Clara, CA, USA), a marker for the intermediate filament glial fibrillary acidic protein. All washing steps were performed three times for 5 min using 0.01M PBS pH 7.4. Sections of 10 µm thickness were first deparaffinised and then rehydrated through a graded series of alcohols. Endogenous peroxidase activity was quenched with 3% hydrogen peroxide for 15 min. Heat mediated antigen retrieval was then performed by microwaving in ethylenediaminetetraacetic acid (EDTA) buffer (pH 8.0). Sections were then incubated in a blocking solution consisting of phosphate buffered saline (PBS) with 0.1% triton and 15% normal goat serum for 1 h prior to incubation with primary antibody. Slides were incubated overnight with anti-aquaporin 4 or anti-GFAP at 4 °C. After washing with PBS, slides were incubated with goat anti-rabbit IgG (1:400; Vector). Sections were washed with PBS and incubated with ABC (Vector) for 1 h. AQP4 or anti-GFAP immunoreactivity was visualised by incubating tissue sections in glucose oxidase diaminobenzidine nickel solution for 5 min. Sections were then washed, dehydrated, cleared in xylene and coverslipped with DPX (Thermo Fisher Scientific, Loughborough, UK).

Tissue sections were examined using an Olympus dot slide microscope. Five non-overlapping regions of interest of 1mm^2^ were captured from the cortical grey matter and underlying white matter from each tissue section. The five regions of interest were adjacent and were selected from similar locations in all cases and the investigator was blinded to the nature of the cases. Image J software (National Institutes of Health) [59] was used to calculate percentage area stained for AQP4 from each image.

### 4.3. Assessment of AQP4 and CAA Severity by Immunofluorescence

The relationship of AQP4 expression to astrocytes and blood vessels in the presence of CAA pathology was qualitatively assessed in all disease states using immunofluorescence and confocal microscopy. Sections were first deparaffinised and then rehydrated through a graded series of alcohols. The following antigen retrieval methods were used, depending on the primary antibody: heat mediated antigen retrieval by microwaving in EDTA buffer (pH 8.0) (for anti-aquaporin 4); 5 min, 1 mg/mL pepsin dissolved in 0.2 M hydrochloric acid at 37 °C (for anti-collagen IV) and 3 min 98 % formic acid (for anti-Aβ 4G8). Sections were then incubated in a blocking solution consisting of PBS with 0.1% Triton and 15% normal goat serum for 1 h prior to incubation with primary antibody. Slides were incubated overnight with the appropriate antibody combination: anti-aquaporin 4 (rabbit polyclonal, 1:200; Santa Cruz), anti-GFAP (mouse monoclonal, 1:100, Proteintech, Manchester, UK), anti-Aβ 4G8 (mouse monoclonal, 1:100, Biolegend, San Diego, CA, USA) and anti-collagen IV (mouse monoclonal, 1:100, Abcam, Cambridge, UK) at 4 °C. After washing with PBS, slides were incubated in appropriate fluorescently labelled secondary antibodies (1:200, (Thermo Fisher Scientific, Loughborough, UK). for 1 h at room temperature. Control experiments were performed by omitting the primary antibodies and by using isotype controls for the monoclonal antibodies. Sections were washed and incubated with Sudan Black (Sigma-Aldrich, Dorset, UK, 1% in 70% alcohol) for 3 min to quench autofluorescence, before being washed and coverslipped with Mowiol (Sigma-Aldrich, Dorset, UK).

Three tissue sections of 10 µm thickness per case were viewed with a Leica TCS SP8 laser scanning confocal microscope and a 63× objective. 660 μm^2^ Z-stack images of cortical grey matter and underlying white matter (×5) were captured from each tissue section. Fluorescent channels were obtained in series, followed by an overlay image of both channels. These images were viewed using Leica Application Suite X software (version 3.4, Leica Microsystems, Milton Keynes, UK).

### 4.4. Statistical Analysis

The investigator was blinded to the true identity of disease state. The data were summarised by disease state using median with lower and upper quartiles, but for cross-referencing purpose with the results in the later analyses, mean with standard deviation were also presented and interpreted. Kruskall–Wallis non-parametric test was performed followed by Mann–Whitney U tests to determine which disease state were significantly different regarding the two outcomes. The parametric equivalent (one-way ANOVA and two-sample t test) were also performed for cross-referencing purpose. Bonferroni correction was applied for all multiple comparisons. The two outcomes were compared within each disease state using the paired sample t test as the difference between the two outcomes was normally distributed. The Braak stages for neurofibrillary pathology data were available only in the old non-demented, moderate and serve CAA brains. The relationship between Braak stage and disease state was assessed using Spearman’s rank correlation. Univariate linear regression analysis was performed on the old non-demented and CAA brains to test for statistical significant differences in percentage area stained for AQP4 separately in grey and in white matter between disease states, Braak stages, age and sex. Adjusted linear regression models were also developed with all 4 covariates, also with disease states and Braak stages only. The same univariate and adjusted regression models were also presented but using the severe CAA group as reference category instead of the old non-demented group to provide results from all three pairwise comparisons. All *p* values presented are referenced at the 5% significance level, and in the case of multiple comparisons between groups, an appropriate new threshold for statistical significance is stated using the Bonferroni correction method. All statistical analyses were performed using IBM SPSS Statistics version 25.

## Figures and Tables

**Table 1:** Demographics of cases used for this study.

Source	Age	Sex	PM Delay /Hrs	Category	Braak Stage	Thal Phase
Edinburgh	50	M	45	Young		
Edinburgh	44	M	47	Young		
Edinburgh	21	M	111	Young		
Edinburgh	29	M	44	Young		
Edinburgh	38	M	49	Young		
Edinburgh	49	M	79	Young		
Edinburgh	32	M	99	Young		
Newcastle	96	F	114	old non-demented	2	3
Newcastle	95	M	21	old non-demented	3	3
Newcastle	83	F	24	old non-demented	1	1
Newcastle	74	M	70	old non-demented	0	0
Newcastle	77	M	46	old non-demented	3	0
Newcastle	89	F	98	old non-demented	3	2
Newcastle	70	M	72	old non-demented	0	1
Newcastle	81	F	31	old non-demented	3	5
Newcastle	76	F	41	old non-demented	2	3
Newcastle	99	F	71	CAA/moderate CAA	6	n/a
Newcastle	63	M	40	CAA/moderate CAA	6	5
Newcastle	78	M	37	CAA/moderate CAA	6	5
Newcastle	90	F	90	CAA/moderate CAA	6	5
Newcastle	87	M	22	CAA/moderate CAA	6	5
Newcastle	81	M	43	CAA/moderate CAA	2	0
Newcastle	86	M	44	CAA/moderate CAA	3	4
Newcastle	62	M	28	CAA/moderate CAA	6	5
Newcastle	78	M	17	CAA/moderate CAA	6	5
Newcastle	73	F	47	CAA/severe CAA	4	3
Newcastle	79	M	13	CAA/severe CAA	3	4
Newcastle	86	F	51	CAA/severe CAA	6	5
Newcastle	86	F	47	CAA/severe CAA	6	5
Newcastle	73	M	7	CAA/severe CAA	6	5
Newcastle	76	F	37	CAA/severe CAA	6	5
Newcastle	78	M	18	CAA/severe CAA	6	5
Newcastle	77	M	78	CAA/severe CAA	6	5
Shefffield	84	F	36	White matter hyperintensity		
Shefffield	78	F	47	White matter hyperintensity		
Shefffield	91	F	35	White matter hyperintensity		
Shefffield	91	F	36	White matter hyperintensity		
Shefffield	89	M	46	White matter hyperintensity		
Shefffield	87	F	17	White matter hyperintensity		

## Supplementary Materials

Supplementary materials can be found at https://www.mdpi.com/1422-0067/21/4/1225/s1.

## Abbreviations

AQP4	Aquaporin 4
CSF	CEREBROSPINAL fluid
CAA	Cerebral amyloid angiopathy
GFAP	Glial fibrillary acidic protein
WMH	White matter hyperintensities
AD	Alzheimer’s disease
