# Supplementary material for: The Pattern of AQP4 Expression in the Ageing Human Brain and in Cerebral Amyloid Angiopathy
Source: Int J Mol Sci. 2020 Feb 12;21(4):1225. doi: 10.3390/ijms21041225 (PMC7072949; doi:10.3390/ijms21041225)
Supplement: Supplementary file 1 [file ijms-21-01225-s001.pdf]

Supplementary Material

a

Disease state	Grey matter N	Grey matter Median (LQ, UQ)	Grey matter Mean (SD)	White matter N	White matter Median (LQ, UQ)	White matter Mean (SD)
	Percentage area stained for AQP4 in…					
**Young (Y)**	7	1.24 (0.32, 1.76)	2.21 (3.43)	7	0.86 (0.48, 1.45)	1.13 (1.06)
**Old non-demented (O)**	9	2.75 (0.58, 3.39)	2.86 (2.48)	9	1.35 (0.05, 6.81)	5.02 (6.63)
**Moderate CAA (M)**	9	4.98 (3.47, 6.43)	5.49 (3.28)	9	0.61 (0.38, 3.84)	2.76 (4.30)
**Severe CAA (S)**	8	1.51 (0.91, 2.79)	2.09 (1.69)	8	1.10 (0.27, 2.83)	2.27 (3.18)
**WMH[1] (W)**	n/a	n/a	n/a	6	1.00 (0.29, 1.52)	1.02 (0.70)

b

Disease state	N	Grey matter – White matter: Mean difference (95% CI)	p
**Young**	7	1.07 (-1.34, 3.49)	0.319
**Old non-demented**	9	-2.16 (-5.66, 1.34)	0.192
**Moderate CAA**	9	2.73 (-1.50, 6.95)	0.175
**Severe CAA**	8	-0.18 (-3.26, 2.91)	0.895
**WMH**	n/a	n/a	n/a

c1

Multiple comparisons between disease state	Grey matter N	Grey matter Median difference (95% CI)	Grey matter p[2]	White matter N	White matter Median difference (95% CI)	White matter p[2]
**O – Y**	16	1.30 (-1.41, 3.15)	0.470	16	0.48 (-0.85, 12.57)	0.758
**M – Y**	16	3.35 (1.03, 5.96)	0.023	16	0.24 (-0.76, 3.40)	0.758
**S – Y**	15	0.45 (-0.85, 1.82)	0.397	15	0.15 (-0.78, 2.25)	0.867
**W – Y**	n/a	n/a	n/a	13	0.12 (-1.10, 1.03)	0.836
**M – O**	18	2.36 (-0.01, 4.76)	0.063	18	-0.14 (-6.43, 1.30)	0.863
**S – O**	17	-1.03 (-2.60, 1.32)	0.815	17	-0.17 (-6.72, 1.70)	0.963
**W – O**	n/a	n/a	n/a	15	-0.35 (-12.93, 1.07)	0.776
**S – M**	17	-3.08 (-5.29, 0.84)	0.021	17	-0.08 (-3.16, 2.24)	0.963
**W – M**	n/a	n/a	n/a	15	-0.10 (-3.45, 0.91)	0.776
**W – S**	n/a	n/a	n/a	14	-0.24 (-2.34, 1.07)	0.755

c2

Multiple comparisons between disease state	Grey matter N	Grey matter Mean difference (95% CI)	Grey matter p[2]	White matter N	White matter Mean difference (95% CI)	White matter p[2]
**O – Y**	16	0.65 (-2.23, 3.53)	0.648	16	3.88 (-0.35, 8.11)	0.071
**M – Y**	16	3.28 (0.40, 6.16)	0.027	16	1.63 (-2.60, 5.86)	0.440
**S – Y**	15	-0.12 (-3.07, 2.84)	0.935	15	1.13 (-3.21, 5.48)	0.599
**W – Y**	n/a	n/a	n/a	13	-0.11 (-4.78, 4.56)	0.961
**M – O**	18	2.63 (-0.60, 5.32)	0.055	18	-2.26 (-6.21, 1.70)	0.255
**S – O**	17	-0.77 (-3.54, 2.01)	0.576	17	-2.75 (-6.83, 1.33)	0.180
**W – O**	n/a	n/a	n/a	15	-4.00 (-8.42, 0.43)	0.075
**S – M**	17	-3.40 (-6.17, -6.24)	0.018	17	-0.49 (-4.57, 3.59)	0.807
**W – M**	n/a	n/a	n/a	15	-1.74 (-6.16, 2.68)	0.429
**W – S**	n/a	n/a	n/a	14	-1.25 (-5.78, 3.28)	0.580

d

Risk factors	Univariate model (N=26) N	Univariate model (N=26) β[3] (95% CI)	Univariate model (N=26) p	Adjusted model 1 (N=26) β[3] (95% CI)	Adjusted model 1 (N=26) p	Adjusted model 2 (N=26) β[3] (95% CI)	Adjusted model 2 (N=26) P
**Dependent variable: Percentage area stained for AQP4 in grey matter**							

**Disease state**			0.030		0.013		0.007
Old non-demented	9	1		1		1	
Moderate CAA	9	2.63 (0.10, 5.17)	0.043	5.38 (1.41, 9.36)	0.010	5.09 (1.55, 8.63)	0.007
Severe CAA	8	-0.77 (-3.38, 1.85)	0.549	2.03 (-1.97, 6.02)	0.302	1.80 (-1.88, 5.49)	0.321
**Braak stage**	26	4.00 (1.34, 6.68)	0.005	-0.78 (-1.63, 0.63)	0.068	-0.74 (-1.52, 0.05)	0.063
**Age (year)**	26	2.76 (-8.06,13.58)	0.603	0.01 (-0.12, 0.15)	0.843		
**Gender**			0.477		0.767		
Male	15	1		1			
Female	11	-0.84 (-3.24, 1.56)	0.477	0.36 (-2.16, 2.89)	0.767		
**Dependent variable: Percentage area stained for AQP4 in white matter**							
**Disease state**			0.481		0.572		0.891
Old non-demented	9	1		1		1	
Moderate CAA	9	-2.26 (-7.11, 2.60)	0.347	3.65 (-3.69, 10.99)	0.312	1.62 (-5.36, 8.60)	0.635
Severe CAA	8	-2.75 (-7.76, 2.26)	0.268	2.19 (-5.18, 9.56)	0.543	1.30 (-5.95, 8.55)	0.713
**Braak stage**	26	-0.90 (-1.81, 0.01)	0.053	-1.42 (-2.98, 0.14)	0.071	-1.16 (-2.70, 0.38)	0.132
**Age (year)**	26	0.02 (-0.21, 0.25)	0.861	-0.06 (-0.31, 0.18)	0.599		
**Gender**			0.194		0.102		
Male	15	1		1			
Female	11	2.58 (-1.40, 6.56)	0.194	3.83 (-0.83, 8.50)	0.102		

e

**From univariate model: Multiple comparisons between disease state**	**Percentage area stained for AQP4 in… Grey matter** N	Mean difference (95% CI)	p[4]	**White matter** N	Mean difference (95% CI)	p[4]
**M – O**	18	2.63 (0.09, 5.17)	0.043	18	-2.26 (-7.11, 2.60)	0.347
**S – O**	17	-0.77 (-3.38, 1.85)	0.549	17	-2.75 (-7.76, 2.26)	0.268
**S – M**	17	-3.40 (-6.01, -0.78)	0.013	17	-0.49 (-5.50, 4.51)	0.840

[1] WMH stands for white matter hyperintensities.
[2] P values are not adjusted for multiple comparisons, significance level is set at p<0.050. After Bonferroni correction for multiple comparisons, significance level should be set at p<0.0083 for grey matter and p<0.0050 for white matter.
[3] β stands for beta coefficient for linear regression model.
[4] P values are not adjusted for multiple comparisons, significance level is set at p<0.050. After Bonferroni correction for multiple comparisons, significance level should be set at p<0.0167 for both grey matter and white matter.

Supplementary Table 1: Statistical analyses. Descriptive statistics summarising AQP4 expression in grey and white matter between disease states. The outcome data were both positively skewed **(a)**. Paired sample t tests showed the difference in percentage stained for AQP4 between grey and white matter in each disease state. In young brains and moderate CAA brains, the expression of AQP4 was higher in grey matter than in white matter. In old non-demented brains and severe CAA brains, the expression of AQP4 was higher in white matter than in grey matter (**b**). Mann-Whitney U tests initially indicated a significant difference (median difference) in AQP4 expression between moderate CAA compared with young and severe CAA in the grey matter; however, this was not the case after a correction for multiple comparisons was done. Similar results were generated using the two-sample t test and were presented with mean differences; these results were mainly for cross-referencing with other analyses in which the mean differences could only be presented **(c)**. Four univariate linear regression analyses (**Univariate model**) on percentage stained for AQP4 in grey and four in white matter using disease state (old non-demented, moderate and severe CAA only), Braak stages for neurofibrillary pathology, age and sex as the independent variables were set up. The results showed a significant relationship between AQP4 expression in grey matter and disease state and also with Braak stage (**d**). With the multiple comparisons between the three disease states, it showed there was a significant difference between AQP4 expression in the grey matter of moderate CAA cases compared to old non-demented, also when compared to severe CAA, however after adjusting for multiple comparisons, these differences were no longer

significant **(e)**. No significant findings from the univariate linear regression on AQP4 expression in the white matter (**d**). Adjusted linear regression model (**Adjusted model 1**) on each outcome showed that after adjusting for Braak stage, age and sex, disease state remained as a significant factor to AQP4 expression in the grey matter, but not in white matter (**d**). Simplified adjusted regression models (**Adjusted model 2**) were explored when only adjusting for Braak stage, similar results to the full model were observed with disease state as a more significant factor for the grey matter outcome (**d**).
